# Arg9 facilitates the translocation and downstream signal inhibition of an anti-HER2 single chain antibody

**DOI:** 10.1186/1756-0500-5-336

**Published:** 2012-07-02

**Authors:** Yi Hu, Chunxia Qiao, Ming Lv, Jiannan Feng, Ming Yu, Beifen Shen, Qiuping Zhang, Yan Li

**Affiliations:** 1 Department of Immunology, School of Basic Medical ScienÎ, Wuhan University, Wuhan, 430071, China; 2 Laboratory of Molecular Immunology, Institute of Basic Medical Sciences, Beijing, 100850, China; 3 Department of Immunology, School of Basic Medical Science, Wuhan University, Donghu Road #185, Wuchang, Wuhan, 430071, China; 4 Laboratory of Molecular Immunology, Institute of Basic Medical Sciences, P.O. Box 130(3), Taiping Road #27, Beijing, 100850, China

**Keywords:** HER2, Single chain antibody, Translocation

## Abstract

**Background:**

HER2 plays a critical role in the pathogenesis of many cancers and is linked to poor prognosis or cancer metastases. Monoclonal antibodies, such as Herceptin against HER2-overexpressing cancers, have showed satisfactory clinical therapeutic effect. However, they have difficulty to surmount obstacles to enter cells or blood–brain barrier.

**Results:**

In this study, a cell-penetrating peptide Arg9 was linked to the C-terminus of anti-HER2 single chain antibody (MIL5scFv). Flow cytometry, confocal microscopy and electron microscopy analysis all revealed that Arg9 peptide facilitated the penetration of MIL5scFv into HER2-negative cell line NIH3T3 and orientate in mitochondria. More interestingly, Western blot assay showed the potential enhanced bioactivity of MIL5scFv-Arg9 in HER2+ cell line SKOV3, indicating that Arg9 could help large molecules (e.g. antibody) to penetrate into cells and therefore enhance its anti-neoplastic function.

**Conclusions:**

Our work represented an attractive by preliminary strategy to enhance the therapeutic effect of existing antibodies by entering cells easier, or more desirable, surmounting the physical barriers, especially in hard-to-reach cancers such as brain metastases cases.

## Background

HER2 is highly expressed in approximately 30% patients with breast or ovarian cancer [1] and represents a potential target for antibody mediated biological therapy against cancers. In clinical investigation, Monoclonal antibody herceptin/trastuzumab has shown satisfactory curative effect in tumour treatment if combined with chemotherapty. However, the effective rate of trastuzumab monotherapy is no more than 25%. [2] One of the reasons is difficulty for high-molecular-weight antibody to surmount the physical barriers to arrive at the destination. Consequently more effective strategies are needed to increase the permeability of antibody drugs against HER2-positive malignancy. Small size substance/antibody such as single chain antibody (scFv) or antigen binding fragment (Fab) is one of the choices based on the report that phage-selected anti-HER2 human scFvs improved antibody induced HER2-mediated endocytosis and displayed strong inhibitory activity on the HER2-overexpressing cancer cell lines, though it is still uncertain whether they have anti-tumour activity *in vivo*. [3]

Currently, cell penetrating peptides (CPPs), including TAT and Arginine rich sequences, [4-6] have been identified to have the capacity to help protein, peptide, PNA, siRNA or DNA to get across the biological membranes into cells. [7,8] For example, β-galactosidase (120 kDa) in its active form can be delivered by TAT to various organs of mice after intraperitoneal injection, such as the lung, liver, spleen and even brain. [9] TAT can also facilitate a single-chain antibody against Bcl-2 to penetrate the mast cell and induce the cell apoptosis. [10] In addition, a synthetic nine-D-arginine oligomer was also reported to efficiently penetrate cell membrane possibly due to its unusual arginine-rich sequence. [11] The advantage of this carrier system is the easy construction of such a small carrying engine. Moreover, it has been reported that Arg9-peptide facilitated the internalization of an anti-CEA immunotoxin and potentiated its specific cytotoxicity to target cells. [12] Therefore, Arg9 was predicted to be useful in the delivery of biological molecules to break through the physiologic barriers and enhance the function of cargo molecules.

In this study, we report a novel fusion protein of Arg9 peptide linked to the C-teminus of the scFv protein derived from an anti-HER2 antibody MIL5 (Chinaese Patent Number: 101591396). This novel Arg9-based delivery system was developed to facilitate MIL5scFv to overcome physical barriers such as cell membranes. Similar to Herceptin, MIL5 inhibits the intrinsic tyrosine kinase activity and down regulates the downstream signalling molecules such as phosphor-Akt (data not shown here). Our experiments showed that this new delivery system could help MIL5scFv overcome the hurdle of the NIH3T3 cell membrane (HER2 negative), and retaining or even improving the specificity of the cargo protein to identify HER2 over-expressing tumour cell line SKOV3. This underlined the highly interesting results of this novel Arg9 based delivery system in terms of facilitating antibody to penetrate the bio-membrane and in the potential design of antibody based therapeutic process against intracellular factors or reaching hard-to-reach targets, such as cerebroma.

## Results

### Expression plasmid construction and protein purification

**Figure 1:**
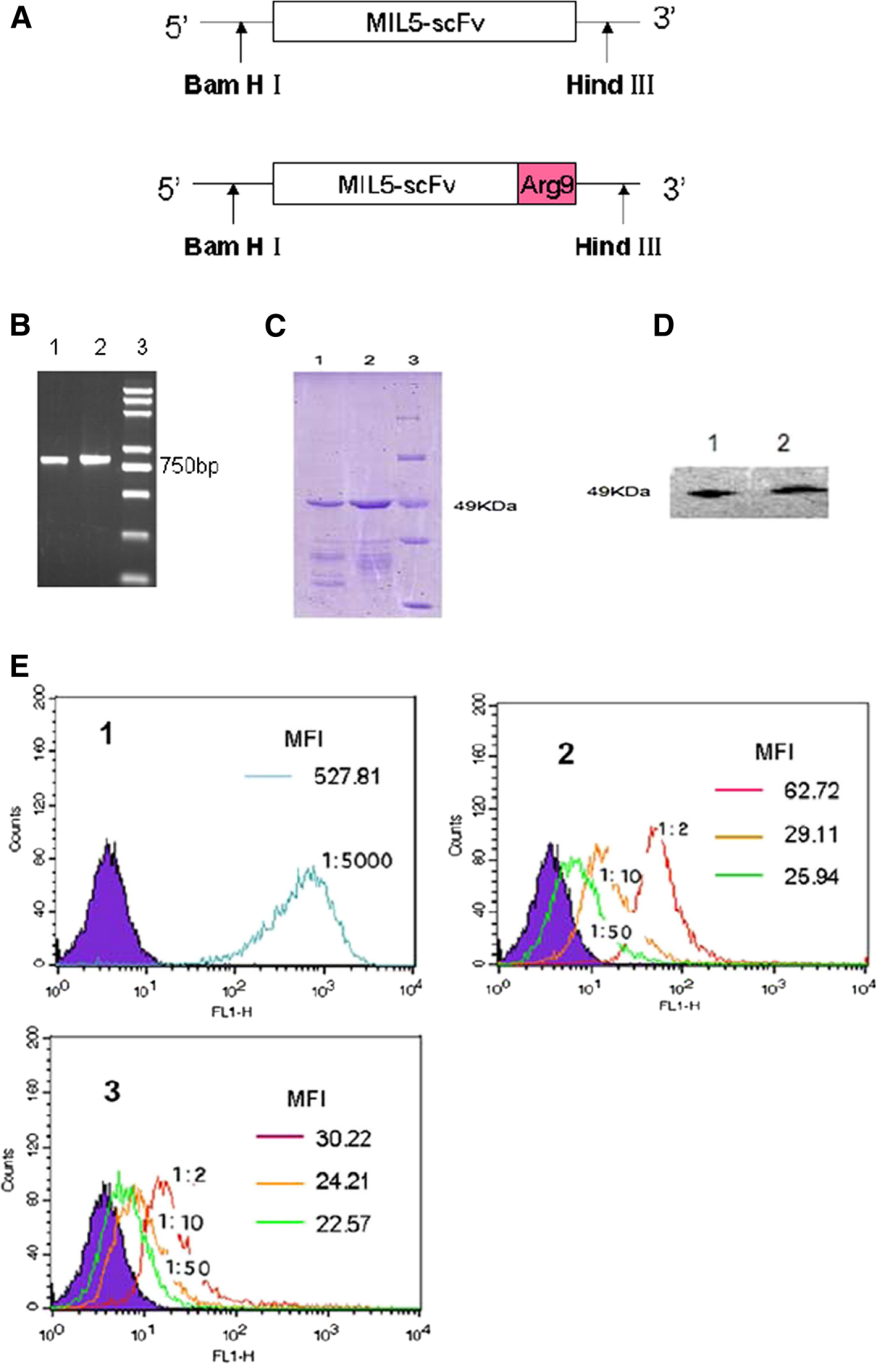
**Expression and identification of fused proteins. A**: Construction of recombinant expression plasmids. pET32a(+)/MIL5scFv and pET32a(+)/MIL5scFv-Arg9. **B**: Gene amplification of scFvs by PCR. Lane 1: MIL5scFv; lane 2: MIL5scFv-Arg9; lane 3: DNA marker DL2000 plus. **C**: SDS-PAGE analysis of purified proteins. Lane 1: MIL5scFv; lane 2: MIL5scFv-Arg9; Lane 3: Protein marker. **D**: Western blot analysis of purified proteins. Lane 1: MIL5scFv; lane 2: MIL5scFv-Arg9. The predicted molecular weight of MIL5scFv-Arg9 was about 49 kDa, which was almost the same with that of MIL5scFv. **E**: Flow cytometry analysis of antigen-binding capacity of purified proteins. Proteins were labelled with FITC in our lab. The F/P quotient of MIL5scFv-FITC is 4.0, while MIL5scFv-Arg9-FITC is 2.7. The cells were treated with labelled proteins on ice for 30 minutes and detected by flow cytometry, respectively. 1: Cells treated with herceptin-FITC as positive control; 2: Cells treated with MIL5scFv-FITC; 3: Cells treated with MIL5scFv-Arg9-FITC. MIL5scFv-FITC and MIL5scFv-Arg9-FITC was diluted as indicated (1:2, 1:10 or 1:50). The binding activity was evaluated with MFI value marked on the right top of each panel and the results showed that they could bind membrane antigen HER2 in a dose-dependent manner. MFI: mean fluorescence intensity. Data are representative of two independent experiments.

To construct the expression plasmid of MIL5scFv fused with Arg9 at C-terminus, an Arg9 peptide encoding DNA was introduced by specific primers (Figure 1.A). The MIL5scFv and MIL5scFv-Arg9 gene were amplified by PCR method and identified by agarose gel electrophoresis (Figure 1B). Then genes were subcloned to pET32a(+) plasmid, respectively, to obtain the expression plasmids pET32a(+)/MIL5scFv-Arg9 and pET32a(+)/MIL5scFv. Proteins were induced by 0.1 mM IPTG, collected from the sonic supernatant and purified by affinity chromatography. The purified protein were electrophoresed in 12% SDS-PAGE (Figure 1C) and further identified by western blot assay (Figure 1D) as the sample proteins based on the predicted molecular weight of 49 kDa.

### MIL5scFv-Arg9 remained the binding capacity to membrane antigen

**Figure 2:**
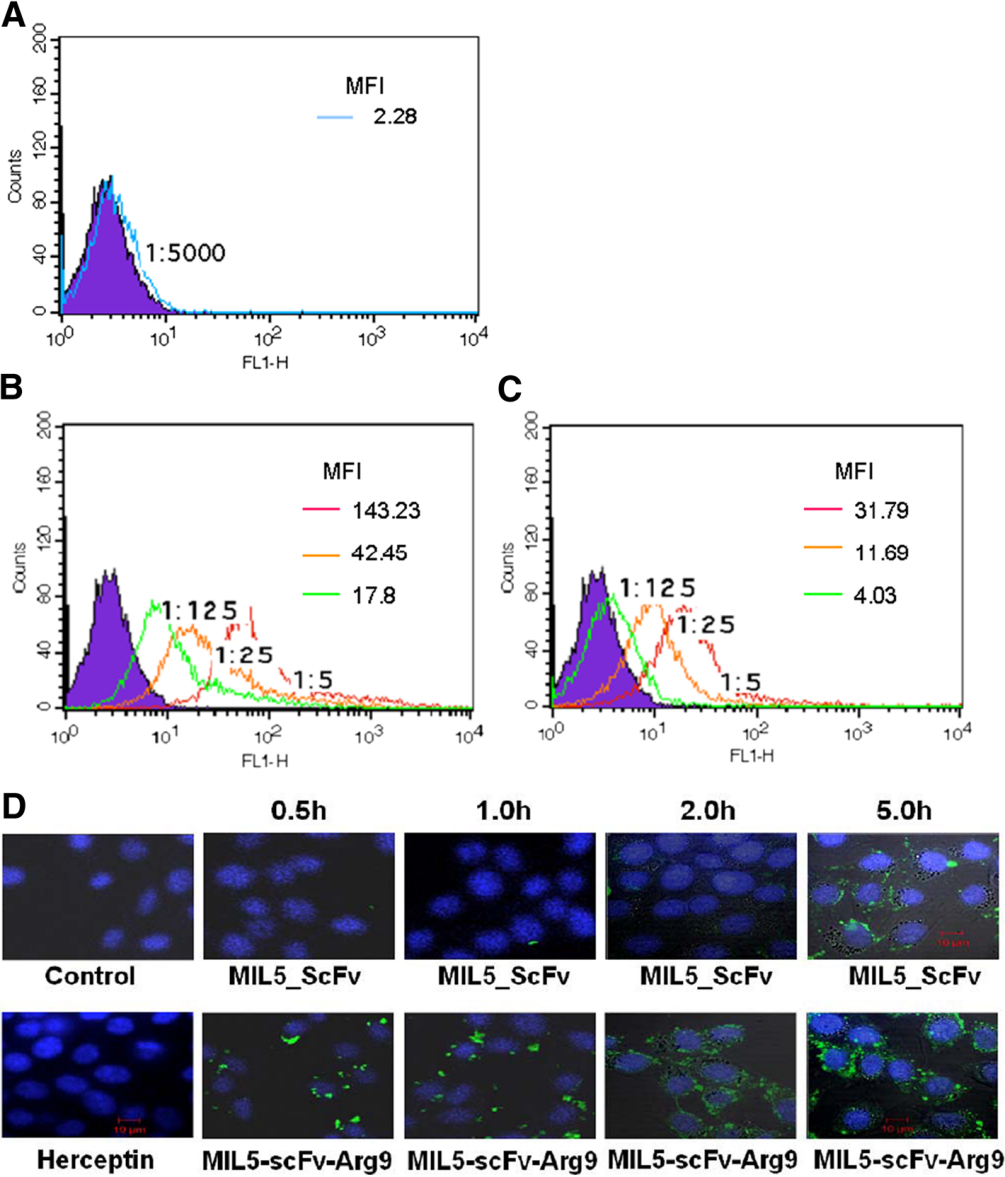
**Flow cytometry analysis and confocal microscopy observation of MIL5scFv-Arg9 to penetrate NIH3T3 cells (HER2 negative).** Cells were incubated with diluted MIL5scFv-Arg9-FITC or MIL5scFv-FITC at 37°C for 2 h and then detected by flow cytometry. **A**: Cells treated with herceptin-FITC as negative control; **B**: Cells treated with MIL5scFv-Arg9-FITC; **C**: Cells treated with MIL5scFv-FITC. The intensity of fluorescence signal was evaluated with MFI value marked on the right top of each panel. According to the fluorescence intensity, Arg9 could help MIL5scFv penetrate NIH3T3 cells. Data are representative of two independent experiments. **D**: Confocal microscopy observation of MIL5scFv-Arg9 to penetrate NIH3T3 cells. Cells were incubated with PBS (Control) or herceptin-FITC as negative control, MIL5scFv-Arg9-FITC (1:10) or MIL5scFv-FITC (1:10) at 37°C and collected consecutively in 0.5 h, 1 h, 2 h and 5 h. The distribution of fluorescence signal in the cells was observed under a laser scanning confocal microscopy. Fluorescent signal could be detected after 0.5 hour in MIL5scFv-Arg9 treated samples, while in MIL5scFv treated samples, weak signal could be seen after 5 hours. Each panel was merged with two photos: FITC conjugated protein (green) and nuclei (blue, cells were stained with Hoechst 33258), which were not shown respectively here.

**Figure 3:**
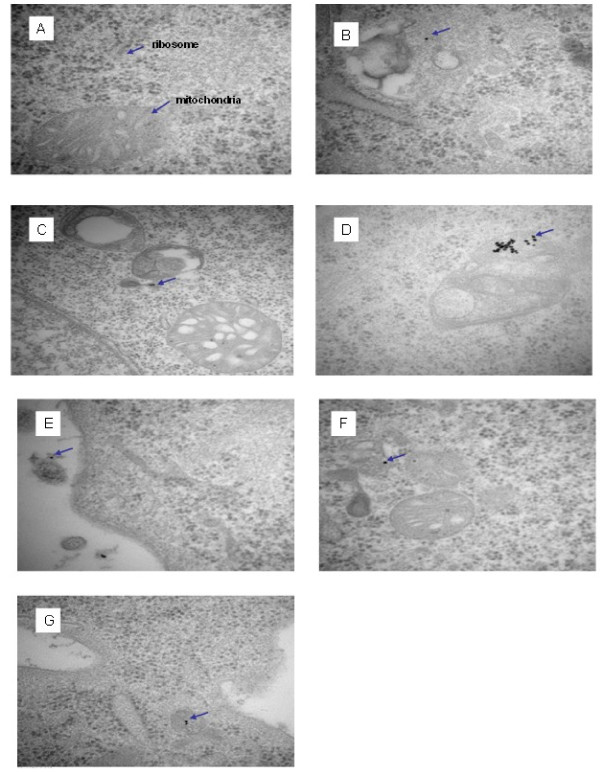
**Transmission electron microscope observation of gold labelled MIL5scFv-Arg9 in NIH3T3 cells.** Cells incubated with PBS (panel **A**), 5 nm colloidal gold labelled with MIL5scFv-Arg9 at 1:40 dilution at 37°C for 1 h, 2 h or 5 h (panel **B**~**D**); 5 nm colloidal gold labelled with MIL5scFv at 1:40 dilution at 37 °C also for 1 h, 2 h or 5 h (panel **E**~**G**), respectively. In one hour, gold particles were found outside or on the membrane of MIL5scFv treated cells (E), while particles can be seen in the endochylema of MIL5scFv-Arg9 treated sample (**B**). After two hours, gold particles could be found inside cells both in MIL5scFv-Arg9 and MIL5scFv treated samples (**C** and **E**), though in figure F they seemed to be near mitochondria. After five hours, the subcellular localization of gold particles in two samples were obviously different, in which MIL5scFv could be seem still in the cytoplasm (**D**), but MIL5scFv-Arg9 aggregated mainly inside of the mitochondria (**G**).

To investigate the capacity to bind membrane HER2 on SKOV3 cells, MIL5scFv and MIL5scFv-Arg9 were conjugated with FITC in our lab (F/P quotient = 4.0 and 2.7, respectively). As shown in Figure 1E, MIL5scFv-Arg9-FITC (Figure 1E-2, at dilutions of 1:2, 1:10 and 1:50) had identical membrane antigen-binding activity compared to MIL5scFv-FITC (Figure 1E-3, at dilutions of 1:2, 1:10 and 1:50). Herceptin-FITC incubated sample was set as positive control (Figure 1E-1, at a dilution of 1:5000). The results suggested that the additional Arg9 peptide did not affect the overall function of the MIL5scFv or its HRE2 biding capacity.

### Arg9 could help cargo protein (MIL5scFv) to penetrate into NIH3T3 cells

To investigate the translocation ability of MIL5scFv-Arg9, we add diluted MIL5scFv-Arg9-FITC or MIL5scFv-FITC (1:5, 1:25 and 1:125) to NIH3T3 cells (membrane HER2 negative). Samples were analyzed by flow cytometry after two hours’ treatment (Figure 2A~C), or collected in different time points for confocal microscopy analysis (Figure 2E). In contrast to MIL5scFv (Figure 2C), the fused Arg9 did facilitate MIL5scFv to penetrate into NIH3T3 cells. This is shown through the fluorescence intensity of the signal shown in Figure 2B. Confocal microscopy analysis again confirmed the enhanced translocation of MIL5scFv-Arg9. After being treated with MIL5scFv and MIL5scFv-Arg9 respectively for 30 minutes, fluorescent signal could be seen only in MIL5scFv-Arg9 treated sample, suggesting that Arg9 could promote large molecule such as MIL5scFv to penetrate bio-membrane. However, when incubation of MIL5scFv prolonged to 5 hours faint fluorescent signal appeared, possibly owing to the non-specific diffusion of MIL5scFv into the weakening cell membranes.

### Arg9 could help cargo protein (MIL5scFv) to translocate in the mitochondria in NIH3T3 cells

In an attempt to identify the possible pathway of translocation and localization of these two proteins, MIL5scFv and MIL5scFv-Arg9 were labelled with 5 nm colloidal gold particles. NIH3T3 cells were incubated with colloidal gold labelled scFvs and observed through transmission electron microscope to analyze the distribution of colloidal gold particles. As shown in Figure 3, in MIL5scFv-Arg9 treated cells, gold particles could be seen inside of the cell membrane after only one hour’s incubation (Figure 3B). Another hour later, MIL5scFv-Arg9 managed to enter mitochondrial membrane, and finally localized specifically in the mitochondrial matrix after five hours’ incubation. In the case of MIL5scFv treated cells, gold particles seemed to be located mainly in cytoplasm (Figure 3E~G) through out the whole process. This showed clearly that Arg9 did facilitate the penetration of cell membrane and mediate the cargo molecule’s translocation into mitochondria in NIH3T3 cells.

### MIL5scFv-Arg9 had stronger inhibitory activity to the expression of phospho-akt in SKOV3 cells

**Figure 4:**
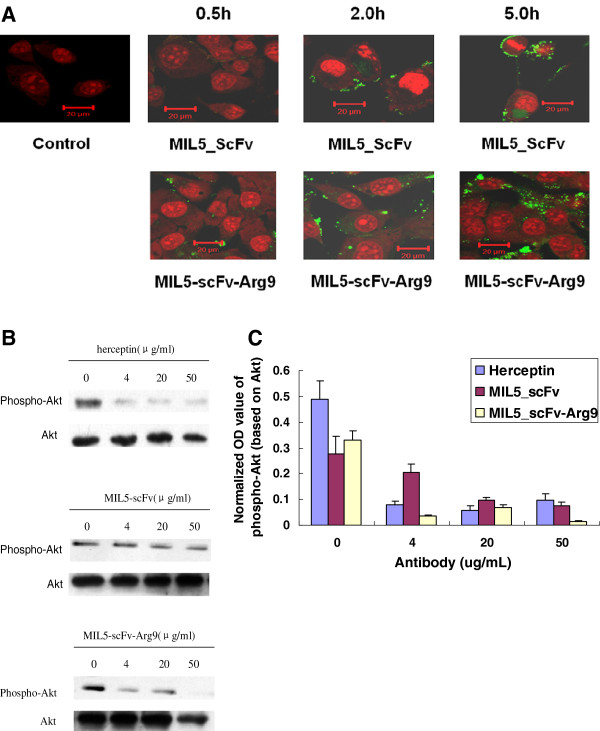
**Enhanced translocation and stronger activity of MIL5scFv-Arg9 to downregulate phospho-Akt in SKOV3 cells (HER2 positive). A**: Confocal microscopy observation of MIL5scFv or MIL5scFv-Arg9 to penetrate SKOV3 cells; Cells were incubated with PBS (Control), MIL5scFv-Arg9-FITC (1:10) or MIL5scFv-FITC (1:10) at 37°C and collected consecutively in 0.5 h, 2 h and 5 h. The distribution of fluorescence signal in the cells was observed under a laser scanning confocal microscopy. Fluorescent signal could be detected after 0.5 hour’ treatment with MIL5scFv-Arg9 contrasting to MIL5scFv treated samples. Each panel was merged with two photos: FITC conjugated protein (green) and nuclei (blue, cells were stained with Hoechst 33258), which were not shown here; **B**: Effect of MIL5scFv or MIL5scFv-Arg9 on the expression of phospho-Akt. SKOV3 cells were treated with diluted MIL5scFv or MIL5scFv-Arg9 (4, 20 or 50 μg/ml, respectively) as indicated, and herceptin treated cells were set as positive control; **C**: Densities of phosphor-Akt were normalized based on densities of Akt shown in Figure 4B, suggesting satisfactory enhanced effect of Arg9 in inhibiting the expression level of phospho-Akt in SKOV3 cells. The data shown are calculated from two independent experiments.

HER2 positive cell line SKOV3 was treated with MIL5scFv-FITC or MIL5scFv-Arg9-FITC. Samples were collected in different time points and analyzed by confocal microscopy (Figure 4A). After 0.5 hour, fluorescent signal could be seen in MIL5scFv-Arg9 treated sample, indicating that Arg9 could help penetrate SKOV3 cells. Furthermore, weaker intracellular fluorescent signal in MIL5scFv treated sample could be seen contrasting to MIL5scFv-Arg9 after 5 hours.

Furthermore, SKOV3 cells were treated and collected for western blot analysis. As shown in Figure 4B, both of MIL5scFv and MIL5scFv-Arg9 treated samples in indicated concentrations remained detectable inhibitory effect on the expression level of phospho-Akt in SKOV3 cells. According to the normalized value of phospho-Akt based on Akt, MIL5scFv-Arg9 was seemed more effective than MIL5scFv. The ratio was about 0.03 in MIL5scFv-Arg9 treated sample contrasting to 0.2 in MIL5scFv treated sample at the concentration of 4 μg/ml, indicating that Arg9 could possibly improve the bio-functional activity of cargo protein (MIL5scFv) to block the HER2 downstream signal transduction.

## Discussion

In this study, a fused gene MIL5scFv-Arg9 was developed through recombinant gene engineering (Figure 1A~B). To obtain the recombinant proteins, we transfected the expression vectors pET-32a(+)/MIL5scFv-Arg9 and pET-32a(+)/MIL5scFv into *E.coli* Rosetta, respectively, and purified proteins were obtained by affinity chromatography from sonic supernatant (Figure 1C~D). Then SKOV3 cells were treated with corresponding purified proteins and then analyzed their binding capacity by flow cytometry method (Figure 1E). Our data showed that short peptide Arg9 did not affect the functional conformation of MIL5scFv, and MIL5scFv-Arg9 kept the identical antigen binding capacity as well as MIL5scFv. Which was consistent with the report that the Arg9 linked to N-terminus of cargo molecule scFv-EGFP could maintain the binding activities to HBsAg and had much better internalization effect. [13]

Arg9 has been reported to have the ability to penetrate the cell membrane. Although the exact mechanism of Arg9 uptake is not yet known, it has been proved to be different from the classic endocytosis pathway. [14] In this study, flow cytometry, confocal microscopy as well as transmission electron microscope analysis were performed successfully to identify the intracellular distribution and location of MIL5scFv-Arg9 in NIH3T3 cells. Our results clearly showed that the fusion protein MIL5scFv-Arg9 could strikingly enhance the cell penetration in a time-dependent manner in contrast to the seemingly weak diffusion of MIL5scFv across the cell membrane after a long treatment for many hours (Figure 2). This diffusion could take place after the bio-membrane was badly weakened by the hour’s long treatment of the MIL5scFv. On the other, it has been reported that Arg6 and Arg8 linked to carbonic anhydrase exhibited the maximum internalization into the macrophage cells and accumulation in the nucleus among the (Arg)_n_(n = 4-16) peptides. [15] The number of arginines required for optimal cell-penetration and the cell localization might depend on the techniques, the cell line used and the characteristic of fused proteins. [16] Therefore, our data demonstrated that Arg9 was an ideal carrier to facilitate MIL5scFv to translocate into endochylema.

The roles of mitochondria in energy production and programmed cell death make this organelle a prime target in the treatment of some disease states. [17] A significant challenge to mitochondrial drug delivery is the impervious structure of the hydrophobic inner membrane. Our data from transmission electron microscope analysis further indicated that MIL5scFv-Arg9 was located mainly in the mitochondria of NIH3T3 cells (Figure 3), while MIL5scFv was only found in endochylema. This suggested that the Arg9 peptide was responsible for the enhanced ability of cell penetration and the specific mitochondrial localization of the fusion protein. Theoretical and experimental studies have revealed the importance of lipophilicity and positive charge in molecules that accumulate in the mitochondria. A modified formula of Arg8 (Cholesteryl-R8) has showed high intracellular selectivity toward mitochondria owing to the guanidinium groups of the arginine residue. [18] In addition, some antioxidants based on penetrating peptide were shown to be located in mitochondrial. [19,20] Thus, Arg9, a molecule of lipophilic nature with strong positive charge as confirmed by Bioinformatic analysis, seemed to be an ideal carrier to facilitate large proteins to enter mitochondria.

Previous studies have also showed that anti-HER2 scFvs selected from phage library enhanced the endocytosis of antigen and showed no growth or signalling impact on HER2-overexpressing cells. [21] However, controversial discoveries declared that the anti-HER2 scFv screened from phage library can inhibit the HER2 signalling, especially the phosphorylation of Akt. [3] In this study, MIL5scFv-Arg9 showed excellent capacity penetrating into SKOV3 cells by the observation of confocal microscopy, and also was identified by western blot analysis to possess stronger effect on inhibiting the expression of phospho-Akt in contrast to MIL5scFv (Figure 4). These indicated that Arg9 could possibly enhance the bio-functional effect of cargo protein *in vitro* and *in vivo.* The single chain antibody against HER2 could hardly play a parallel role of the whole antibody; however, with the help of Arg9, the fusion protein might be able to assert a satisfactory inhibitory effect of tumour cell proliferation or survival through the HER2-Akt signalling pathway.

## Conclusions

Our data demonstrated that Arg9 peptide retained and even enhanced the function of cargo molecule (MIL5scFv) with regard to the antigen binding and downstream signal transduction in SKOV3 cells. Arg9 could obviously enhance the penetration of MIL5scFv into NIH3T3 cells, and make the way for the cargo molecules to be located mainly in mitochondria. When fused to specific antibody or other macromolecules, Arg9 shows the potential of an interesting and useful carrier for mitochondrial delivery of specific antibody. In addition, the *in vitro* cell penetrating activity of Arg9 should be further exploited for the study of diverse endocellular biological events. Although our work indicates the hypothesis and possibility of using Arg9 to deliver antibody therapeutics in hard-to-reach cancers such as brain metastases cases *in vivo*, [22] further studies should be conducted to investigate its mechanism of penetration and other biological effects induced by such a unique carrier.

## Methods

### Reagents

pET-32a(+) vector was from Promega (Madison, WI, USA); IPTG was from Sigma(USA); Ni^2+^–nitrilotriacetic acid sepharose was from Chinese academy of sciences (China); FITC was from Sigma; herceptin was purchased from Genentech (USA); anti-Akt and anti-phospho-Akt (Ser 473) were from Cell Signaling technology (USA); RGS·His HRP conjugate (HRP_anti-His, HRP conjugated anti-His antibody) was purchased from QIAGEN (Germany); horseradish peroxidase-conjugated secondary antibody was from Zhongshan Goldenbridge (China); MIL5scFv and MIL5scFv-Arg9 were prepared in our laboratory.

### Cell lines and culture conditions

Human ovarian cancer cell line SKOV3 (high expression level of HER2), and immortalized cell line NIH3T3 were obtained from ATCC. The cells were cultivated in DMEM (high level of glucose) supplemented with 100u/ml of penicillin, 100u/ml of streptomycin and 10% fetal bovine serum (FBS). All the cells were incubated in a humidified incubator (Thermo, America) at 37°C with 5% CO_2_.

### Construction of expression plasmids

MIL5 was an anti-HER2 antibody prepared in our laboratory. The variable genes of heavy and light chains were amplified with specific oligo-primers and PCR method. MIL5scFv gene was then synthesized using a (SG4)3 linker between light and heavy sequences. After amplification, products were electrophoresed in 1.2% agarose gels. MIL5scFv and MIL5scFv-Arg9 genes, in which Arg9 peptide was fused to the carboxyl-terminus of MIL5scFv as described in Figure 1, were subcloned into BamHI-Hind III-digested pET-32a(+) vector (Promega, Madison, WI, USA) to construct the expression plasmids pET-32a(+)/MIL5scFv and pET-32a(+)/MIL5scFv-Arg9. The sequences of expression plasmids were further confirmed by DNA sequencing analysis.

The sequence of sense primer was 5′-CGCGGATCCGATATCGTGATGACCC-3′, containing a BamHIsite. The sequence of antisense primer was 5′-CCCAAGCTTTTACCGTCTCCGCCTGCGTCTCCTGCGTCTGGAGCTCACGGTCACCAGGG-3′, introducing the Arg9 peptide gene and a Hind III restriction site, or 5′-CCCAAGCTTTTACCGTCTCCGCCTGCGTCTCC-3′, only introducing a Hind III restriction site.

### Prokaryotic expression and purification of recombinant proteins

*E. coli* Rosetta cells were transfected with expression plasmids and cultivated in 2 × YT medium supplemented with ampicillin to a final concentration of 100 μg/ml at 37 °C to a OD_600_ value of 0.5–1.0, respectively, followed by another 12 hours’ shaking at 27°C after adding 0.1 mM isopropyl-β-D-thiogalactopyranoside (IPTG). Cells were collected by centrifugation, resuspended and lysed by sonication at 4°C in binding buffer (20 mM PB supplemented with 300 mM NaCl, pH 8.0). After centrifugation, supernatant was immediately loaded onto a Ni^2+^–nitrilotriacetic acid sepharose affinity column (Chinese academy of sciences, China). After being washed with 10 volumes of a binding buffer, the column was loaded with diluted imidazole (10 mM, 30 mM, 50 mM, 200 mM, and 500 mM) in 20 mM PB, pH 8.0. The fusion proteins were collected and desalted using Millipore column (MWCO, 10000). The concentrations of purified proteins were calculated by ultraviolet spectrophotometer.

### SDS-PAGE and western blot analysis

Purified proteins were mixed with loading buffer to perform SDS-PAGE using 12% gel and detected with coomassie staining solution. The other resolved proteins were transferred from the gel onto a nitrocellulose membrane. The membrane was blocked with 5% milk for 1 h at room temperature and then incubated with HRP_anti-His at a dilution of 1:5000 in 5% milk for an hour’s incubation at room temperature. After being washed three times with TBS-T, the membrane was detected by ECL and autoradiography.

To verify the expression levels of phospho-Akt, SKOV3 cells were seeded in six-well plates and grown for attachment for 12 h. Then the cells were serum-starved for another 12 h and then treated with herceptin, MIL5scFv or MIL5scFv-Arg9 at different concentrations for 24 h. Cells were washed once with cold PBS and lysed in M2 buffer (1 mol/L Tris-cl, 25% NP-40, 5 mol/L NaCl, 0.5 mol/L EDTA, 0.5 mol/L EGTA, 10 μg/ml aprotinin, 1 mol/L DTT, 10 mol/L PNPP, 1 mol/L Na_3_VO_4_) for 3 min on ice, and collected with cell scrape. Cells were then centrifuged at 12000 rpm for 15 min at 4°C. The supernatant was collected for Western blot analysis. The nitrocellulose membranes were incubated overnight at 4°C with anti-Akt or anti-phospho-Akt (Ser 473) monoclonal antibody (Cell Signaling technology, USA) and then incubated with horseradish peroxidase-conjugated secondary antibody (Zhongshan Goldenbridge, China). Other detection did as described above. The experiments were carried out two times.

### Flow cytometry assay

SKOV3 cells were harvested through centrifugation and washed once with cold PBS (pH7.4) containing 2% FBS. Cells were incubated in FITC labelled MIL5scFv (MIL5scFv-FITC) or MIL5scFv-Arg9 (MIL5scFv-Arg9-FITC) at different concentrations (1:2, 1:5, 1:10 dilution) or herceptin (1:5000) for 30 min at room temperature. Cells were then washed twice and analyzed by a FACScan cytometer (Becton Dickinson, Mountain View, CA).

NIH3T3 cells were harvested, collected and incubated with FITC labelled herceptin (herceptin-FITC, 1:5000), MIL5scFv-FITC or MIL5scFv-Arg9-FITC at different concentrations (1:5, 1:25, 1:125) for 2 h at 37°C. Samples were then analyzed as described above for evaluating the ability of Arg9 in helping cargo molecule penetrate cell membranes. The experiments were carried out two times.

### Confocal microscopy observation

Cells were grown on Glass Bottom Dish (3x10^5^cells/well) in complete medium for 24 hours at 37°C, treated with MIL5scFv-FITC or MIL5scFv-Arg9-FITC for 1 h, 2 h or 5 h and then fixed with 4% paraformaldehyde. After permeabilization with 0.1% Triton in PBS containing 2% FBS, the cells were exposed to Hoechst 33258 and resuspended in 100 μl PBS. Samples were observed and photographed by laser scanning confocal microscope using the Zeiss LSM 410 confocal laser system connected to a Zeiss Axiovert 135 M microscope with 40_/1.2 C-Apochromat water immersion lens (Zeiss, Jena, Germany). Green fluorescence of FITC was excited with argon laser (488 nm for excitation with 525 nm pass barrier filter for radioactive wave). The similar results were obtained in two times experiments.

### Transmission electron microscopy observation

Colloidal gold (CG, 5 nm) conjugated MIL5scFv and MIL5scFv-Arg9 were prepared with redox method. NIH3T3 cells were incubated for 1 h, 2 h or 5 h at 37 °C with 5 nm CG-conjugated particles in serum free DMEM medium (1:40 dilution) and fixed with 3% glutaraldehyde at 4 °C for 2 h, followed by being fixed with 1% osmium tetroxide.After dehydration, embedding, section, and staining treatment, transmission electron microscopy (Philips CM120) was used for observation.

## Abbreviations

CPPs: Cell penetrating peptides; Arg9: Nine-arginine; scFv: Single chain antibody; IPTG: Isopropyl-β-D-thiogalactopyranoside; FITC: Fluorescein isothiocyanate; MFI: Mean fluorescence intensity; OD: Optical density.

## Competing interests

The authors declare that they have no competing interests.

## Authors’ contributions

H carried out protein expression, transmission electron microscope assay and wrote the manuscript; Q carried out protein purification, confocal microscopy observation and revised the manuscript; Lv participated in protein labelling. F synthesized DNA sequence; Y carried out FACS assays; S participated in the design of the study; Z designed and conceived the coordination of the study; Li participated in the design of the study and helped to draft the manuscript. All authors read and approved the final manuscript.

## Availability of supporting data

We agree that our data are available in an open access repository, and we agree to “BioMed Central Open Access license agreement”. We have taken due care to ensure the integrity of the article.

We warrant that the article is original, has not been formally published in any other peer-reviewed journal, is not under consideration by any other journal and does not infringe any existing copyright or any other third party rights;

We are the sole authors of the article and have full authority to enter into this agreement and in granting rights to BioMed Central are not in breach of any other obligation.
